# Phytochemical Profiling, Antioxidant Activity, Food Preservation, and Insecticidal Properties of *Origanum syriacum* and *Cymbopogon winterianus* Extracts

**DOI:** 10.3390/foods14081347

**Published:** 2025-04-14

**Authors:** Marwa Rammal, Maya Kara, Adnan Badran, Chaden Haidar, Hawraa Zahreddine, Hussein Bassal, Mikhael Bechelany, Mohammad H. El-Dakdouki, Akram Hijazi

**Affiliations:** 1 Department of Food and Technology Studies, Faculty of Agronomy, Lebanese University, Beirut P.O. Box 146404, Lebanon; marwa.rammal.1@ul.edu.lb (M.R.); mayakaraa13@gmail.com (M.K.); chaden.haidar@iul.edu.lb (C.H.); hbassal@hotmail.com (H.B.); 2 Department of Nutrition, University of Petra, Amman P.O. Box 961343, Jordan; abadran@uop.edu.jo; 3 Departement of Hospitality, Faculty of Tourism, Islamic University of Lebanon, Khaldeh P.O. Box 30014, Lebanon; akram.hijazi@ul.edu.lb; 4 Nutrition and Food Science, School of Arts and Sciences, Lebanese International University, Beirut P.O. Box 146404, Lebanon; hawraa.zahreddine@liu.edu.lb; 5 Institut Européen des Membranes (IEM), École Nationale Supérieure de Chimie de Montpellier (ENSCM), Centre National de la Recherche Scientifique (CNRS), Place Eugène Bataillon, UMR-5635, University of Montpellier, 34095 Montpellier, France; 6 Functional Materials Group, Gulf University for Science and Technology, Mubarak Al-Abdullah P.O. Box 32093, Kuwait; 7 Department of Chemistry, Faculty of Science, Beirut Arab University, Riad El Solh, Beirut P.O. Box 11-5020, Lebanon; 8 Plateforme de Recherche et D’analyse en Sciences de L’environnement (EDST-PRASE), Beirut P.O. Box 6573/14, Lebanon

**Keywords:** Syrian oregano, Java citronella, antioxidant activity, food preservative, flatbread, insecticide

## Abstract

This study examines the phytochemical composition, antioxidant, antifungal, and insecticidal properties of *Origanum syriacum* (Syrian oregano plant) and *Cymbopogon wimterianus* (Java citronella plant) extracts. Their potential applications in food preservation and pest control are explored based on their bioactive properties. The phytochemical screening indicated a rich presence of secondary metabolites in the extract. The hydrodistillation of plant leaves resulted in an extraction yield of 4.3% Syrian oregano essential oil. The major component of the essential oil was carvacrol (79.30%). The Syrian oregano ethanolic extract contained 110.674 ± 1.842 mg GAE/g total phenols and 52.57 ± 0.086 mg RE/g total flavonoids, and exhibited a high antioxidant activity with a half-maximal inhibitory concentration (IC_50_) equal to 168.28 μg/mL. Flatbread was prepared with additions of Syrian oregano and Java citronella powders, followed by analysis of moisture content, visual appearance, and sensory characteristics. The results showed that the powders of Syrian oregano and Java citronella have promising food preservative effects. These findings were supported by a significant decrease in fungal growth in several samples and a shelf life extension of one day. The inclusion of a 2% mixture of Syrian oregano and Java citronella powder in the flatbread resulted in the sample receiving the highest overall acceptability mark from consumers, while also extending its shelf life. To assess the insecticidal activity, weevils (*Sitophilus granarius* L.) were exposed to Syrian oregano and Java citronella essential oils. The insecticidal activity was at its peak when Syrian oregano and Java citronella essential oils were combined resulting in 7% lethal dose (LD_50_) towards grain weevils. Future research should focus on optimizing extraction methods, evaluating long-term storage effects, and assessing the broader applicability of these extracts in various food products and agricultural settings.

## 1. Introduction

The food industry is a constantly evolving system that must adapt to meet the ever-changing demands and preferences of consumers. It is crucial to consider a variety of quality-related factors throughout the manufacturing and marketing of new food products, including food safety, nutritional value, shelf life, and sensory appeal. Newly added ingredients must enhance the quality of products while also considering sensory analysis, which is crucial for ensuring food acceptability [1]. Around the world, researchers are seeking innovative and safe methods to preserve food. In addition to the considerable time and financial resources needed to develop and approve new synthetic preservatives, synthetic food additives are going through a challenging time right now, especially considering the public backlash against them. There is an increasing pressure on food manufacturers to either fully eliminate the synthetic hazardous preservatives or employ natural alternatives in order to maintain or extend a product’s shelf life [2]. In this perspective, incorporating medicinal plants into food items is an excellent and appealing approach to create functional foods given the abundance of phytochemicals in plant-based extracts, which are particularly valued for their beneficial health effects [3]. The increasing interest in medicinal plants as natural substitutes to artificial additives in food is driven by consumer demand for healthier products. While the shelf life of food may be shortened if synthetic antioxidants are reduced or eliminated, plant-based alternatives possess antimicrobial, anti-inflammatory, bactericidal, insecticidal, antiviral, antifungal, and preservative properties, making them promising candidates for extending food shelf life. However, further studies are still required on sensory properties, sources of antioxidants and antimicrobials, maximizing extraction efficiency, and extending food shelf life [3].

In this context, two medicinal plants were of interest in this study: *Origanum syriacum* L., commonly referred to as Syrian oregano, Lebanese oregano, bible hyssop, and za’atar in Arabic (Lamiaceae family), and *Cymbopogon winterianus* Jowitt, also known as citronella grass and Java citronella (Poaceae family). *O. syriacum* is an aromatic perennial herb originating from the Eastern Mediterranean, western Asia, and southern Europe and is cultivated all over the world. This crop has been used in traditional medicine for addressing various illnesses, especially in Arab nations [4]. However, it is now frequently used in culinary arts intended for human consumption [5], such as in the formulation of the Middle Eastern za’atar spice blend [6]. The essential oil of oregano is an important product of the plant [7]. It is characterized by several interesting properties, such as antioxidant, antimicrobial, insecticidal, and antifungal, and has a variety of usages spanning from food preservation and flavoring to utilization in fragrance and pharmaceutical industries [8]. *C. winterianus* is a highly stress-tolerant, aromatic herbaceous plant [9] that grows in tropical and subtropical areas across Asia (especially Indonesia), Africa, and the Americas [10]. It is a widely popular product across these continents [11]. Citronella grass is a natural, safe antioxidant rich phenols, terpenes, nitrogen compounds, and ascorbic acid, rendering it a healthy product for human consumption [12]. This plant has various applications in medicine, food, and agriculture, including insect repellent, antifungal agent, natural preservatives, and fragrance [11]. The essential oil of Java citronella is also an essential product of the plant [12,13] and its effectiveness as a natural repellent varies based on factors like concentration, formulation, and environmental conditions. Lebanon, like most Arab countries, is renowned for its daily bread intake. Standard commercial bread, however, could be deficient in key nutrients, such as antioxidants. A fresh approach to adding antioxidants to bread is to use functional ingredients with considerable antioxidant content [13]. Also, the short shelf life of bread has led to significant waste and the risk of foodborne illnesses. Therefore, maintaining quality in wheat-based products with a high moisture content remains a challenge and a key concern in the food industry that strives toward preventing bread deterioration [14]. Oregano has been studied as a potential natural preservative in food products such as fresh salmon [15], bread, cheese [16], and fermented cooked sausages [17] with overall sensory acceptance.

This study aimed to assess the chemical composition of extracts and essential oil from Lebanese *Origanum syriacum*. Its primary objective was to evaluate the effectiveness of Syrian oregano and a blend of Syrian oregano and Java citronella in powdered form as food preservatives, focusing on their physical, organoleptic, and microbiological properties in flatbread. Additionally, the study examined the insecticidal effects of the essential oils from these plants against granary weevils. The findings contribute to the existing literature on both Syrian oregano and Java citronella in Lebanon and beyond.

## 2. Materials and Methods

### 2.1. Equipment

The employed Gas Chromatography–Mass Spectrometer (GC-MS) included an Agilent 6890N (Santa Clara, CA, USA) Network Gas Chromatograph equipped with an Agilent 19091S-433HP-5MS column (30 m × 0.25 mm × 0.25 µm dimensions). A grinder (Compact 2100, IKA-Werke GmbH & Co. KG, Staufen, Germany), clean plastic containers, nylon bags, rotary evaporator Rotovap (Evaporateur extracteur rotatif VV 200 Heidolph S (Instruments GmbH & Co. KG, Schwabach, Germany; SN: 129515157)), refractometer type ABBE (serial number A8493302), Vortex (XH-B), and Hitachi U-2900 UV-Vis spectrophotometer (High-Technologies Corporation, Tokyo, Japan) were the other instruments utilized in the current study.

### 2.2. Chemicals and Reagents

Chemicals and reagents utilized in this study were used as received from the vendor. Ferric chloride, 95% ethanol, acetone, chloroform, concentrated sulfuric acid, concentrated hydrochloric acid, glacial acetic acid, and copper acetate were purchased from VWR. DPPH, Fehling A and B solutions, sodium carbonate, and ninhydrin were acquired from GPR. Folin–Ciocalteu reagent (10%), sodium hydroxide, and potassium hydroxide were obtained from UNI-CHEM. Gallic acid, aluminum chloride, and potassium ferricyanide (K_3_(Fe(CN)_6_)) were purchased from AnalaR. Distilled water was used in all experiments. The raw materials used in the production of flatbread were whole wheat flour type 85 (crown flour mills, Lebanon), salt, dry yeast, and water.

### 2.3. Powder Preparation

**Figure 1:**
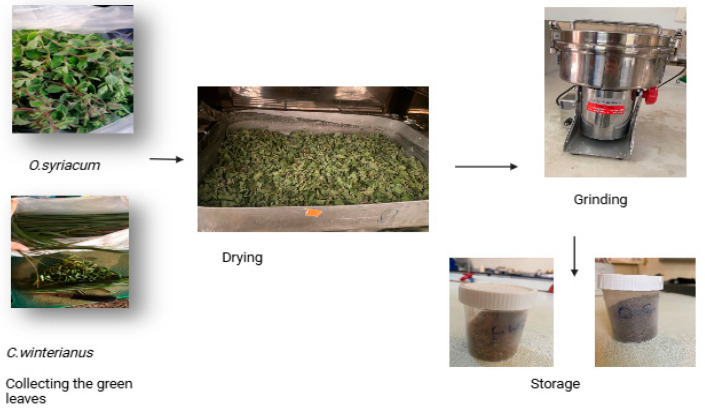
Preparation of plant-based powder for storage and use.

The aerial parts of Syrian oregano and Java citronella plants were collected from the towns of Khiam and Blat, respectively, in Southern Lebanon during the period between March and June 2023. The fresh leaves of both plants underwent thorough washing with distilled water before being air-dried in the shade at room temperature for a month. Upon complete drying, the peels of each plant were separately pulverized into fine powder (Figure 1). These powders were subsequently stored in sterilized plastic containers, covered with nylon, and placed in a desiccator to protect them from moisture.

### 2.4. Extract Preparation

The technique of maceration was employed for preparing the plant’s extracts. For the preparation of the oregano aqueous extract, 30 g of oregano was combined with 250 mL of distilled water. Likewise, another 30 g of oregano was mixed with 250 mL of 95% ethanol to prepare the oregano ethanolic extract. Both containers were sealed and stored in darkness for 24 h (extraction time) at room temperature (20–25 °C). Subsequently, the resulting extracts were filtered using filter paper and a Buchner funnel. Both filtrates were then concentrated on a rotary evaporator (Rotovap) set at 40 °C. The concentrated extracts were then stored at −80 °C, after which they were processed on a lyophilization machine. The obtained powders were stored in sanitized plastic containers, covered with nylon, and placed in a desiccator to avoid moisture.

### 2.5. Essential Oil Extraction

**Figure 2:**
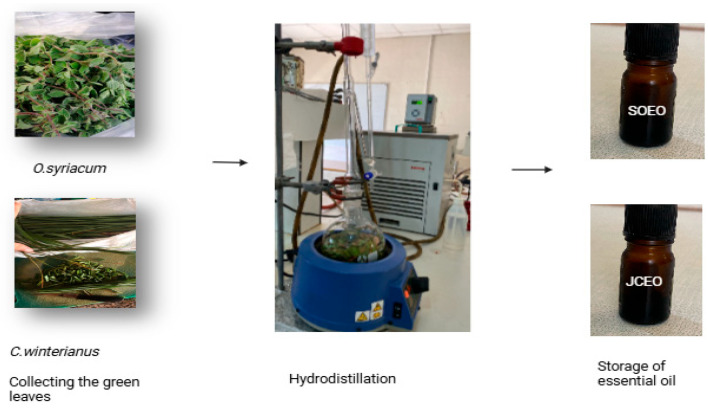
Extraction of essential oil from leaves using hydrodistillation.

The extraction of Syrian oregano essential oil (SOEO) was achieved by hydrodistillation using a Clevenger type apparatus (3.3 Boro Glass Distillation Unit, Glassco Labs, India) over a duration of 1.5 to 2 h, until no essential oil (EO) was obtained. Next, 50 g of the fresh leaves was placed in the flask with 500 mL distilled water and heated. The vapors were condensed in the cooler, and the oil was separated from the water due to a difference in density (Figure 2). The recovered SOEO was then stored in an amber glass bottle at 4 °C. The yield (*R*) of EO is the ratio of the volume of essential oil and plant material mass and is expressed as a percentage (%).

(1) $$R=\frac{V_{HE}}{M_{VS}}\times 100$$

where $V_{HE}$ is the volume of EO (mL) and $M_{VS}$ is the mass of plant material (g).

The same procedure was followed for the extraction of Java citronella essential oil (JCEO).

#### 2.5.1. Relative Density of the Essential Oil

The relative density (ρ) of SOEO, expressed in g/cm^3^, was determined in accordance with the ISO 279:1998 standard method by first measuring the density (ρ20) [18], which is the mass of a unit volume (1 mL) of the essential oil at 20 °C, and then applying Equation (2):

(2) $$\rho =\frac{1}{relative\;density\;of\;water\;at\;20{}^{\circ}C}\times \rho 20=1.00180\times \rho 20$$

#### 2.5.2. Refractive Index of the Essential Oil

The refractive index (RI) of Syrian oregano essential oil (SOEO) was measured using an Abbe refractometer at a controlled temperature of 20 ± 0.5 °C. The instrument was calibrated prior to measurement using distilled water (20 °C) and verified with a standard reference material of known refractive index. Approximately 2–3 drops of the SOEO sample were placed on the cleaned prism surface of the refractometer, ensuring no air bubbles were present. The prism assembly was then closed, and the sample was allowed to equilibrate for 1–2 min to reach thermal stability. The refractive index reading was taken once a stable value was observed. Measurements were performed in triplicate to ensure accuracy and reproducibility [19].

#### 2.5.3. Chromatographic Analysis of the Essential Oil

A Gas Chromatography-Mass Spectrometry (GC-MS) analysis was employed to identify the compounds present in the extracted SOEO and JCEO. A volume of 1 µL of the essential oil was introduced into the GC inlet, maintaining a column flow rate of 1.3 mL/min. Initially, the column temperature was set to 325 °C, while the injection temperature was set to 280 °C. The oven temperature was gradually increased from 65 °C to 450 °C at a rate of 3 °C/min. Subsequently, the detector (MS) scanning proceeded for 45 min. Individual components were identified by comparing their mass spectra with references in the GC-MS library (NIST05a.L).

### 2.6. Qualitative Analysis–Phytochemical Screening

The qualitative tests outlined in Table 1 were utilized to determine the chemical composition of Syrian oregano aqueous and ethanolic extracts.

### 2.7. Quantitative Analysis

#### 2.7.1. Total Phenolic Content (TPC)

The total phenolic content of the oregano ethanolic extract was determined using the Folin–Ciocalteu reagent method with slight modifications [27]. Briefly, 100 μL of the extract was mixed with 1000 μL of 1/10 diluted Folin–Ciocalteu reagent and incubated for 6 min in the dark. Then, 1000 μL of 7% Na₂CO₃ was added, and the mixture was vortexed and incubated for 30 min in the dark at room temperature. The absorbance of the resulting blue solution was measured at 765 nm using a Hitachi U-2900 UV-Vis spectrophotometer (U-2900, Hitachi High-Technologies Corporation, Tokyo, Japan). The total phenolic content was calculated as milligrams of gallic acid equivalent (GAE) per gram of dry extract, using a calibration curve of known gallic acid concentrations. The total phenolic content was calculated using Equation (3).

(3) $$TPC=\frac{\mathrm{GAE}\;\times \;V\;\times \;D}{W}$$

where GAE represents the gallic acid equivalence (mg/mL) and is determined by correlating optical density (O.D.) values with the gallic acid standard curve (y = 0.0046x + 0.0259; R^2^ = 0.9963), where V denotes the volume of extract (mL), D indicates the dilution factor, and W signifies the weight (g) of the pure plant extract.

#### 2.7.2. Total Flavonoid Content (TFC)

The total flavonoid content of the oregano ethanolic extract was determined using the aluminum chloride method with slight modifications [28]. In this method, 1000 μL of the extract was mixed with 1000 μL of 2% methanolic aluminum chloride, followed by vortexing and incubation for 30 min in the dark at room temperature. Absorbance was measured at 415 nm using a Hitachi U-2900 UV-Vis spectrophotometer. The results were expressed as micrograms of rutin equivalent (RE) per gram of dry extract, calculated using Equation (4).

(4) $$TFC=\frac{\mathrm{RE}\;\times \;V\;\times \;D}{m}$$

where RE represents the rutin equivalent (μg/mL) and is determined by correlating optical density (O.D.) values with the rutin standard curve (y = 0.0182x + 0.0169; R^2^ = 0.9988), where V denotes the total volume of the sample (mL), D indicates the dilution factor, and m represents the weight of the sample (g).

### 2.8. Antioxidant Activity: DPPH Assay

The antioxidant activity of Syrian oregano ethanolic extract was assessed using the 2,2′-Diphenyl-1-picrylhydrazyl (DPPH) radical scavenging method with slight adjustments to the protocol by Rammal et al. (2012) [29]. Different concentrations (0.1, 0.2, 0.4, 0.6, 0.8, and 1 mg/mL) of the extract were mixed with 1 mL of DPPH solution, and the control and blank solutions were also prepared. The mixtures were vortexed and incubated in the dark for 30 min at room temperature. Absorbance was then measured at 517 nm using a Hitachi U-2900 UV-Vis spectrophotometer to evaluate antioxidant activity. The percentage scavenging of the DPPH radicals by the different samples was calculated using Equation (5).

(5) $$Percent\;of\;inhibition=\frac{absorbance\;of\;control-absorbance\;of\;the\;extract}{absorbance\;of\;control}\times 100$$

Next, the IC_50_, which refers to the half-maximal inhibitory concentration, was determined. The percentage of DPPH scavenging was graphed against concentration, and a linear regression was employed to determine the IC_50_ value (μg/mL). Experiments were conducted in triplicate.

### 2.9. Food Preservation

#### 2.9.1. Flatbread Preparation Method

To assess the impact of oregano powder on flatbread quality, dried powdered oregano leaves were incorporated at various concentrations: 0.5%, 1%, 1.5%, 2%, 2.5%, and 3% of the total amount of whole wheat flour. All ingredients were combined for approximately 5 min at medium speed using a dough mixer (Moulinex). The resulting dough samples were left to ferment at room temperature for 2 h. Subsequently, the flatbread samples were baked at 115 ± 5 °C for 3 min each in a preheated oven. Following baking, the loaves were allowed to cool at room temperature (22 ± 2 °C) for 2 h. Once cooled, the flatbread samples were placed in nylon bags, labeled with sample numbers, sample codes, and production dates, and stored at room temperature in a well-ventilated environment [30]. Similarly, to assess the impact of a mixture of oregano and citronella powder on flatbread quality, dried powdered oregano and citronella leaves were added at concentrations ranging from 0.5% to 3% of the total amount of whole wheat flour. The same baking procedure was followed for these samples. A control flatbread, where no plant powder was included in the recipe, was employed for comparison purposes. The quantities of these ingredients remained consistent across all samples. Visual assessments of the samples were conducted over an 8-day period to detect any signs of mold. Images were captured on day 1, day 4, and day 8 after baking. The sample codes used are detailed in Table 2. Experiments were performed in triplicate.

#### 2.9.2. Flatbread Moisture Content

The moisture content of the flatbread was assessed using the mass loss on drying method. Each flatbread sample underwent oven drying at 105 ± 5 °C until a consistent mass was achieved. The samples were weighed before and after drying. This procedure was conducted on day 1 and repeated on day 4 and day 8, with triplicate measurements taken each time. The moisture content (MC) was determined using the following formula:

$$MC=\frac{m_{i}-m_{f}}{m_{i}}\times 100$$

where *m_i_* represents the initial mass of the flatbread sample before drying (in grams), and *m_f_* denotes the final mass of the flatbread sample after drying (in grams).

To analyze the difference in moisture content (MC) between the control and the other samples on day 1, day 4, and day 8, a factorial analysis of variance (ANOVA) was conducted using STATISTICA 64.

#### 2.9.3. Sensory Evaluation

A hedonic sensory analysis of 4 flatbread samples and the control sample were conducted at the Lebanese University, Faculty of Agricultural Engineering and Veterinary Medicine at pilot scale. The analysis was performed with 60 panelists between the ages of 18 and 60, regarding several organoleptic characteristics (appearance, texture, odor, color, flavor, mouthfeel, after taste, and overall acceptability), using a 9-point hedonic scale (1: extremely dislike; 9: extremely like), labeled “Dislike Extremely” at the left end, “Like Extremely” at the right end and “Neither Like nor Dislike” at the center of the scale. All samples were served in the shape of small triangles, placed in identical containers, and code labeled using three-digit, randomly generated numbers. A glass of water was also served to each panelist to overcome the carry-over effects. A one-way ANOVA was applied using STATISTICA 64 to analyze the overall acceptability of flatbread samples.

### 2.10. Insecticidal Activity

#### 2.10.1. Preparation of Dishes

Adult grain weevils (*Sitophilus granarius*) found on freekeh wheat were incubated and left to reproduce and replicate in a convenient environment to be used in this experiment. Ten weevils were placed per Petri dish, which was firmly closed afterwards. The Petri dishes were labeled with the sample code and the date of the experiment on the opposing end.

#### 2.10.2. Preparation of Solutions

Solutions of different concentrations (2%, 3%, 5%, 7%, 10%) of SOEO or mixture of SOEO and JCEO were prepared as shown in Table 3 in 10 test tubes that were cleaned thoroughly and left to dry at room temperature. The solutions contained different types and percentages of EO mixed with a volume of ethanol that adds up to 1 mL final volume for each solution. Each tube was sealed well in a nylon fold to prevent the evaporation of essential oil. Each solution was placed in the corresponding labeled Petri dish containing 10 weevils and left for 2 days at room temperature. Images were taken at intervals of 30 min, 6 h, 9 h, 12 h, 24 h, and 36 h after the start of the experiment.

## 3. Results and Discussion

### 3.1. Essential Oil Yield and Characteristics

The SOEO obtained through hydro-distillation in this study is characterized by pale yellow color, a density (ρ) of 0.93, and a refractive index of 1.502. There is no official standard regarding the characteristics of SOEO. It is a mobile liquid, clear with a pale yellowish color and a strong characteristic odor, like that of za’atar. Given their high density and refractive index, the SOEO that was retrieved as of good grade [31].

The obtained yield of SOEO was 4.3%, and it is higher than that of *O. syriacum* of different soil types from South of Lebanon, which varied from 0.1% to 4.1%. Indeed, the yield of SOEO is affected by various factors such as geographical location, cultivation practices, harvesting season, soil conditions, extraction techniques, temperature, post-harvest procedures, and other relevant factors [7].

### 3.2. Essential Oil Chemical Composition

The GC-MS analysis of SOEO identified eight compounds (Table 4), with carvacrol (79.3%) as the dominant component, aligning with previous studies from Lebanon (78.4%) [32], Egypt (76.6%) [33], and Syria (69.8%) [6]. The low thymol content (0.47%) confirms SOEO as a carvacrol chemotype, being similar to *O. syriacum* oils from Qartaba and Achkout (Lebanon). The carvacrol-to-thymol ratio in SOEO is consistent with Mediterranean oregano species, though it differs from some European variants, which contain higher thymol levels. Carvacrol is widely recognized for its antimicrobial, antioxidant, and anti-inflammatory properties, disrupting bacterial membranes and preventing lipid oxidation, making SOEO a potential natural preservative. Additionally, citronellal, a minor compound in SOEO, enhances its antifungal and insect-repelling capabilities. Environmental factors such as altitude, temperature, and soil composition strongly influence oregano’s chemotypic variability, with warmer climates favoring higher carvacrol biosynthesis [34]. Given these bioactive properties, SOEO could be valuable in food preservation, antimicrobial treatments, and pharmaceutical formulations, though future research should explore seasonal and environmental effects on its composition and efficacy.

The results for the qualitative chemical analysis of Syrian oregano in the current study are in accordance with that of *O. syriacum* collected from Southern Lebanon confirming the presence of the following metabolites in ethanolic extract: tannins, resins, phenols, flavonoids, quinones, sterols and steroids, cardiac glycosides, and terpenoids [5]. Phytochemical screenings seek to detect diverse secondary metabolites in plants, with variations depending on the type of the extraction solvent employed. Ethanol is widely acknowledged for its ability to dissolve a wide array of phytochemicals, while water may selectively extract specific compounds [35]. Other intrinsic and extrinsic factors may also influence the phytochemical profile of plant extracts. These factors include plant maturity, geographical location, environmental conditions, and extraction methods.

### 3.3. Qualitative Analysis–Phytochemical Screening

Both aqueous and ethanolic extracts of Syrian oregano were found to be relatively rich in primary and secondary metabolites (marked by +), as listed in Table 5.

### 3.4. Quantitative Study

The total phenolic content (TPC) and total flavonoid content (TFC) for Syrian oregano were measured at 110.674 ± 1.842 mg GAE/g of dry extract and 52.571 ± 0.086 mg RE/g of dry extract, respectively. Both values are lower than those reported in a study conducted in Egypt, where the TPC was 148 mg GAE/g and the TFC was 81.87 mg RE/g of dry extract [36]. These differences in TPC and TFC values could be attributed to several factors including environmental conditions, agricultural practices and genetic factors. The TPC and TFC levels found in this study are nevertheless relatively high, indicating that Syrian oregano has strong antioxidant potential despite the lower values observed.

**Figure 3:**
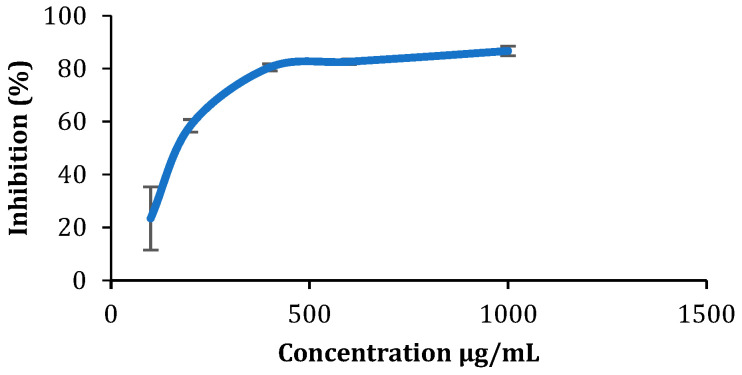
Percentage of DPPH radical scavenging as a function of Syrian oregano ethanolic extract concentration.

Syrian oregano ethanolic extract expressed a concentration-dependent antioxidant activity (Figure 3). The inhibition percentage increased proportionally with concentration until it reached a maximum of 86.71% inhibitory antioxidant activity at 1000 μg/mL. Since IC_50_ values are inversely proportional to antioxidant activity, the highest antioxidant activity is associated with the lowest IC_50_ concentration. Oregano extract exhibited an antioxidant activity with IC_50_ equals to 180.95 μg/mL, indicating robust radical scavenging capabilities. These results are similar to those of a study that was conducted on *O. Syriacum* collected from South of Lebanon and resulted in IC_50_ values between 150 and 200 μg/mL for ethanolic extract [5].

### 3.5. Oregano and Java Citronella Powders as Food Preservatives in Flatbread

#### 3.5.1. Visual Analysis over Time

On days 1, 2, and 3, no mold was visually detected on any of the flatbread samples. On day 4, all samples exhibited mold growth, with the exception of the sample supplemented with a 2% combination of Syrian oregano and Java citronella powder (2C+O). By day 5, all samples demonstrated signs of mold growth.

**Figure 4:**
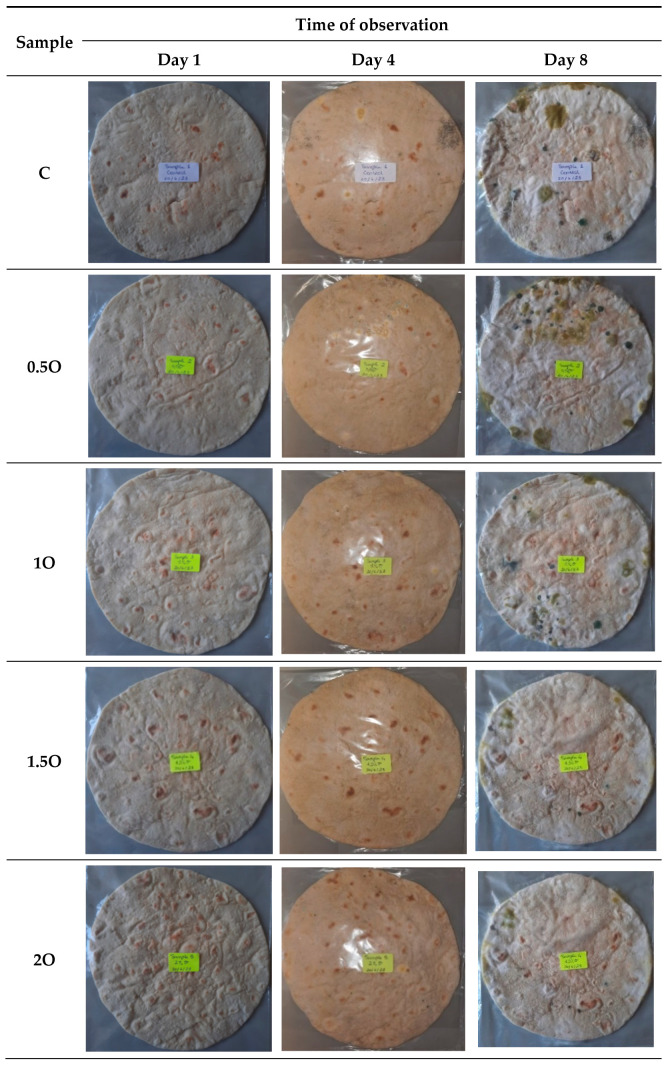

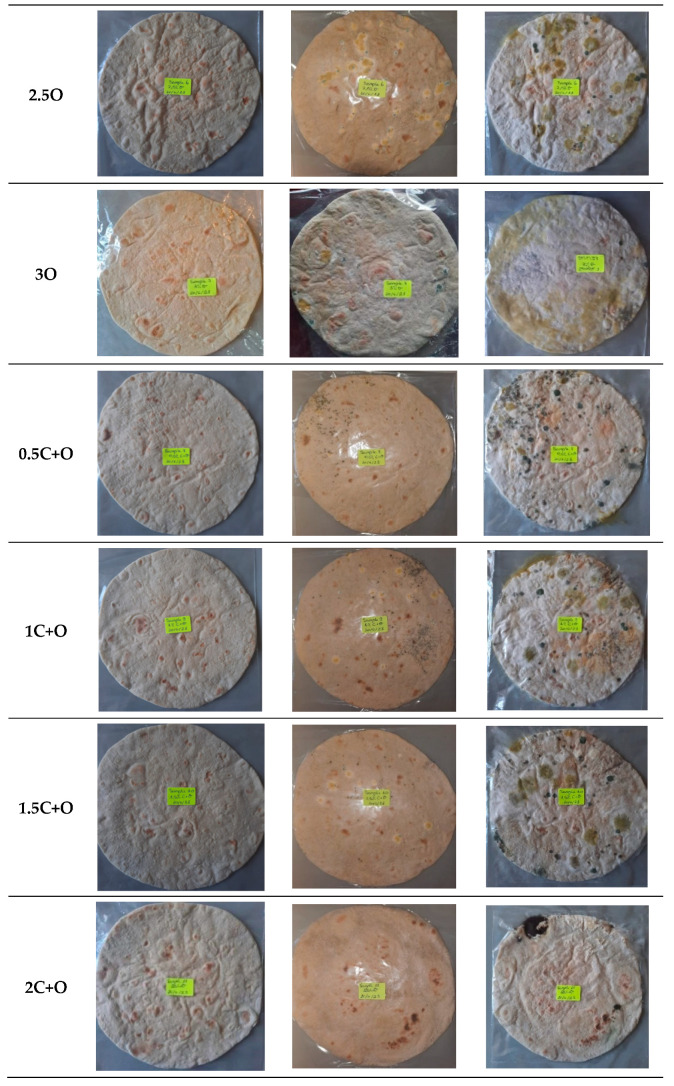

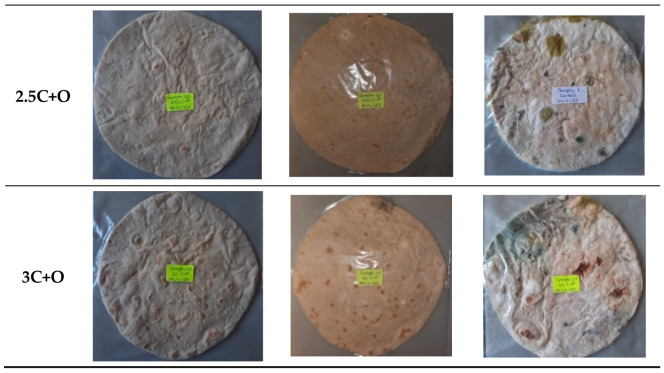
Flatbread samples on day 1, day 4, and day 8.

According to the visual analysis of flatbread samples (Figure 4), the following results were obtained: Concerning the addition of oregano powder alone, the concentrations of 0.5%, 1%, 1.5%, and 2% exhibited a reduction in fungal growth during storage at room temperature compared to the control sample, with 1% and 1.5% as the concentrations having the best effect. However, concentrations of 2.5% and 3% resulted in a higher fungal growth compared to the control sample, excluding these concentrations from potential use as preservatives. Concerning the oregano and citronella powder mixture, the concentrations of 2%, 2.5%, and 3% led to a reduction in mold growth during storage at room temperature compared to the control sample containing no powder, with 2% and 2.5% C+O offering the best observed results. The remaining concentrations, i.e., 0.5%, 1%, and 1.5%, showed no positive effect on reducing microbial development compared to the control. Among all samples, the highest reduction in fungal growth in the flatbread during storage was observed when the flatbread was supplemented with 2% oregano and citronella powder mix. In fact, it is the only sample where the shelf life of the product was prolonged by a day, making the shelf life 4 days compared to a shelf life of 3 days for the remaining samples, including the control. These results can be compared with that of a study showing a strong mold-inhibiting quality in bread products supplemented with oregano tincture and orange peel powder [16]. Also, another study revealed that tomato pomace powder had a positive effect on the physicochemical properties of flatbread [37]. Similar observations were reported when ground coffee beans with known antioxidant properties were added to bread [13].

The observed antifungal properties of these plant powders can be attributed to their bioactive compounds. Carvacrol, the predominant component in Syrian oregano essential oil, exerts its antimicrobial effects by integrating into microbial cell membranes, leading to structural disruptions and increased permeability, which ultimately results in cell death. Similarly, citronellal, a major constituent of Java citronella oil, targets ergosterol, a vital component of fungal cell membranes, disrupting membrane integrity and inhibiting fungal growth [38]. Higher levels of oregano in flatbread did not translate to increased antifungal activity. Several factors can contribute to this observation. It has been reported that higher concentrations of the bioactive volatile components in the oregano powder can lead to its rapid release from the flatbread matrix at elevated temperatures, thereby reducing their long-term effectiveness. On the other hand, lower levels of the volatile components result in its slow and controlled release, providing sustained antifungal activity. In addition, higher amounts of the oregano can affect the water activity of bread, possibly making it susceptible to fungal growth. Therefore, it is critical to identify the minimal effective concentration of oregano that preserves the desired antifungal properties without adversely affecting the characteristics of flatbread [39,40].

#### 3.5.2. Flatbread Moisture Content

**Figure 5:**
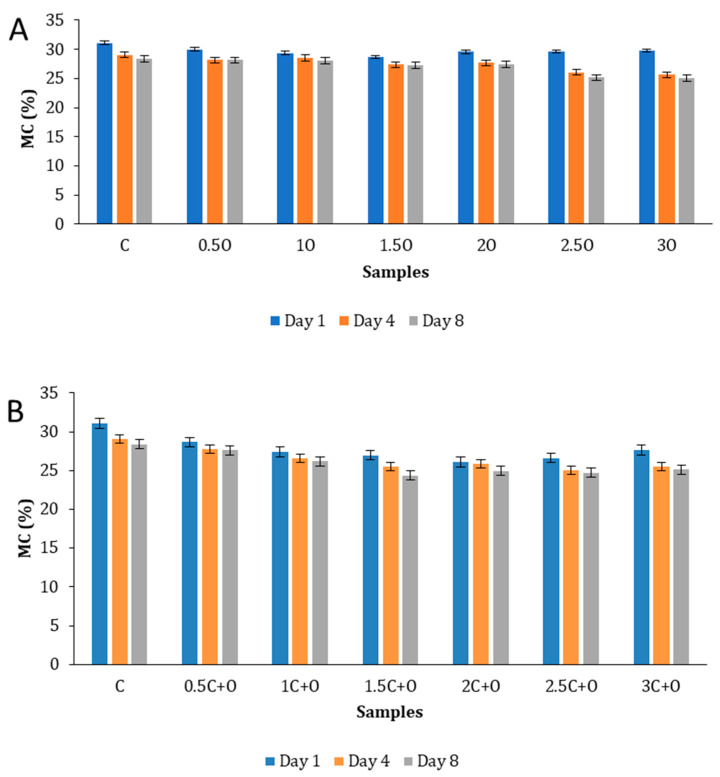
Moisture content (MC) variation between day 1, day 4, and day 8. (**A**) Flatbread samples supplemented with Syrian oregano powder; (**B**) flatbread samples supplemented with Syrian oregano and Java citronella powder. The values are represented as the mean ± standard deviation (SD).

Figure 5 displays the moisture content of flatbread enriched with plant powder on days 1, 4, and 8. Flatbreads have a MC superior to 20% and can retain moisture of over 35% [41]. This was confirmed in our samples, where the MC was 26% on day 1 (Figure 5). Samples containing a concentration of 1, 1.5, 2, 2.5, and 3% oregano and citronella powder (C+O) had an initial MC significantly inferior to that of the control sample C. This can be linked to the decrease in and retardation of fungal growth in certain cases (Figure 4) since microbes develop faster in higher MC environments. However, the proliferation of microbes in food cannot be attributed solely to moisture content [42]. On day 4 and day 8, the MC of samples containing 2.5 and 3% oregano, as well as 1.5, 2, 2.5, and 3% C+O, was significantly inferior to that of the control sample on each day. The type of packaging used and the relative humidity of the storage room may also influence the MC.

#### 3.5.3. Flatbread Sensory Analysis

**Figure 6:**
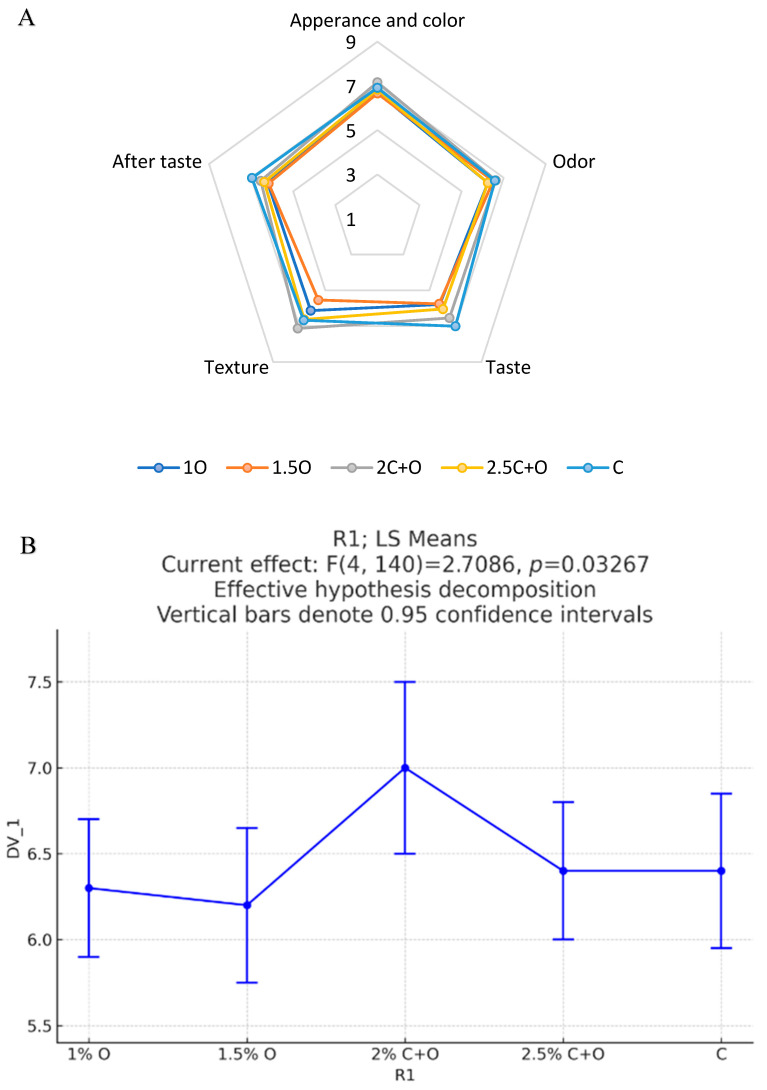
Hedonic sensory analysis for the five flatbread samples tested. (**A**) Radar plot illustrating the hedonic profile; (**B**) statistical analysis of the overall acceptability.

All samples are relatively similar in most organoleptic parameters (Figure 6A), primarily odor, as well as appearance and color parameters, where the 2C+O sample scored the highest, followed by aftertaste where the control sample scored the highest. The biggest differences were in texture and taste. The control flatbread has the best perceived taste and the 2C+O flatbread has the best perceived texture (elasticity). The 1.5O flatbread sample scored the lowest in both parameters. Overall, all samples are considered acceptable with scores superior to 5.7 over 9.

In terms of the overall acceptability of the samples (Figure 6B), there was no notable distinction between the control sample, the 2.5C+O sample, and the 1O sample (*p* > 0.05). However, a significant difference in overall acceptability was reported between the 2C+O and 1.5O samples with the highest and lowest acceptability, respectively (*p* < 0.05). All samples are considered acceptable with a score superior to 6.1 over 9.

Based on these results, the control sample and the sample supplemented with a 2% concentration of oregano and citronella powder mixture (2C+O) were the most favorable according to the panelists. These findings align with a study examining the impact of oregano herb on dough rheology and bread quality where the findings indicated that oregano can be added to bread at a quantity of up to 2% without significantly altering its baking and sensory characteristics or shortening its shelf life [43]. Furthermore, the addition of an oregano and citronella powder mixture of a quantity ranging between 2 and 2.5% also yielded promising results in the current study.

### 3.6. Essential Oils for Pest Control

#### 3.6.1. Visual Assessment over Time

**Figure 7:**
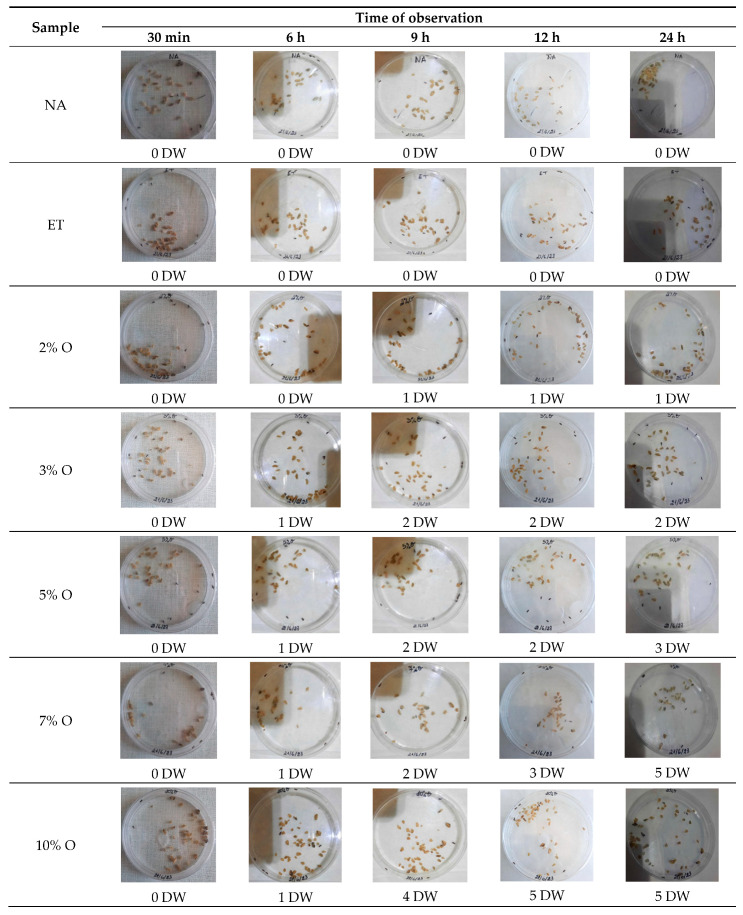

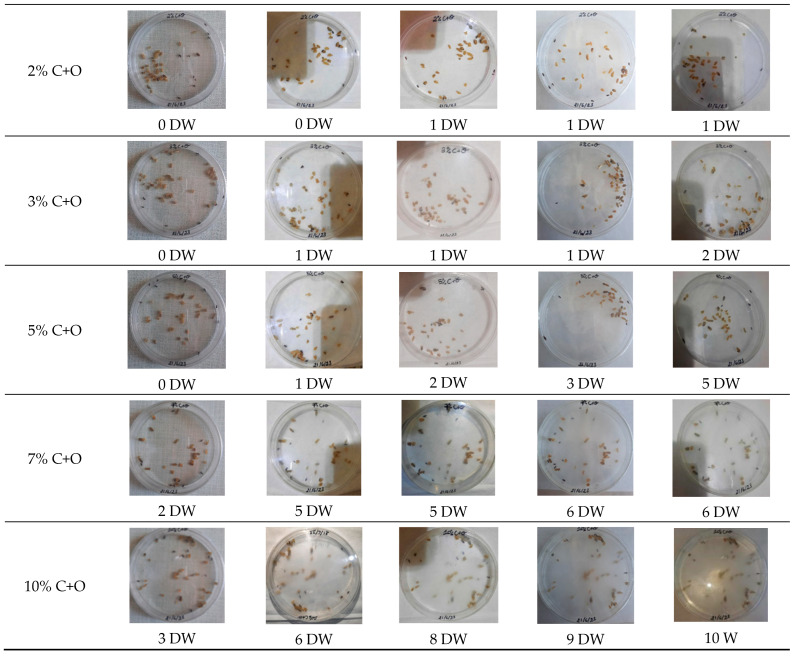
Visual counting of dead weevils after exposure to different concentrations of SOEO and SOEO+JCEO. DW: Dead weevils.

Figure 7 illustrates the number of dead weevils after several periods of time when different concentrations of SOEO or a mixture of SOEO and JCEO diluted in ethanol were added. In addition, the potential effect of ethanol (ET) alone on the weevils’ viability was tested. A control (NA) sample, where neither essential oil nor ethanol was added, represented the negative control. All samples initially contained 10 grain weevils.

#### 3.6.2. Determination of Lethal Time 50 (LT_50_)

**Figure 8:**
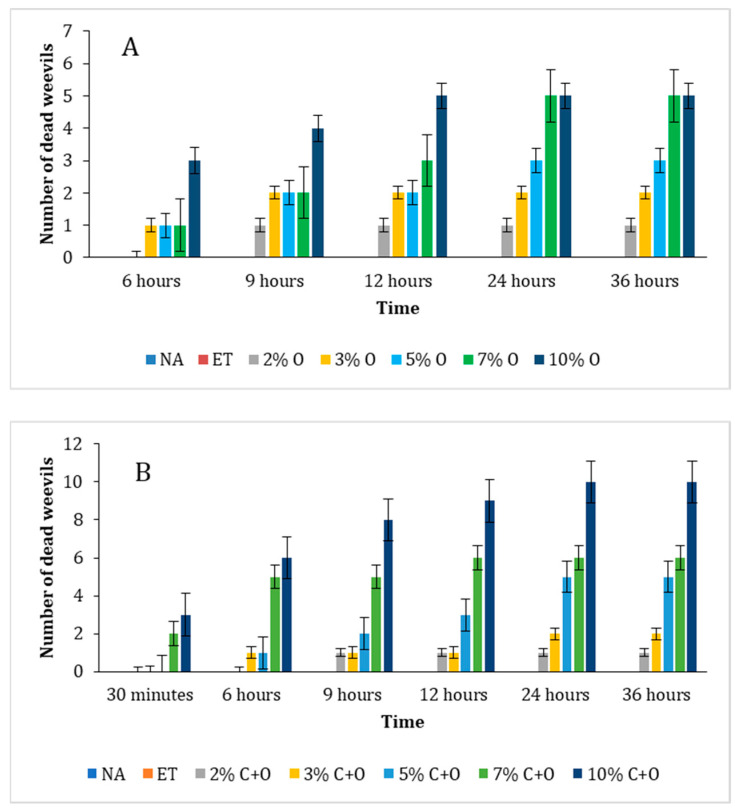
Impact of treatments on weevil mortality over time. (**A**) Impact of SOEO; (**B**) impact of SOEO + JCEO mixture.

The following charts (Figure 8) are used to determine the LT_50_ (the time after which 50% of the insects die following the application of different solutions to evaluate the efficacy of the administered treatments, and results are summarized in Table 6. It is evident from Figure 8A that 50% of the weevils were killed by the oregano treatments at 7% and 10% concentrations after 24 h and 12 h, respectively. Figure 8B illustrates that when oregano was combined with citronella, 50% of the weevils were killed at 5%, 7%, and 10% concentrations after 24 h, 6 h, and less than 6 h, respectively. Notably, the 10% oregano-citronella combination demonstrated the most rapid and potent effect on weevil mortality compared to the other treatments. Some studies investigating insects’ mortality extended observations to 48, 72, and 96 h to assess delayed or sublethal effects, evaluate long-term efficacy, and ensure that the late onset mortality is accounted for [44,45]. In the current study, observations were reported over 36 h as the goal was to assess acute and rapid effect of the oregano and oregano–citronella mixtures on the mortality of weevils.

#### 3.6.3. Determination of Lethal Dose 50 (LD_50_)

**Figure 9:**
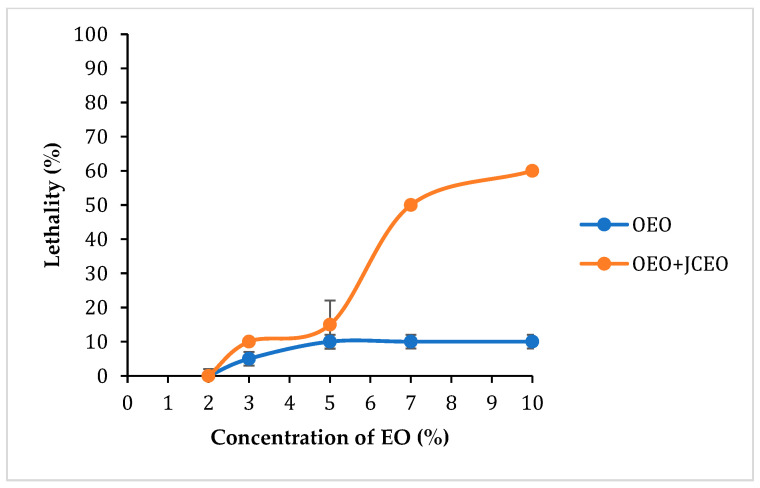
Mortality of weevils at 6 h following treatment with the essential oil.

Figure 9 shows the variation in the percentage of dead weevils as a function of the essential oil concentration. LD_50_, defined as the dosage of solution causing 50% mortality of weevils after 6 h, was extrapolated to evaluate the efficacy of the treatments administered. It is evident that only the combination treatment achieved 50% mortality of weevils after 6 h at 7% concentration which subsequently represented the LD_50_ for the SOEO+JCEO treatment. This value could not be established for the SOEO treatment alone. These values are listed in Table 6.

The insecticidal effect is dependent on two key factors, which are the concentration of the added EO and the time of exposure. As per the visual assessment (Figure 7), 30 min after the beginning of treatment, all oregano doses and the 2%, 3%, and 5% C+O combinations showed no lethal effect. However, 7% and 10% C+O showed 20% and 30% lethality, respectively. After 6 h, 2% O and 2% C+O showed no lethal effect, while 3% O, 5% O, 7% O, 10% O, 3% C+O, and 5% C+O displayed similar lethality of 10%. Notably, 7% C+O and 10% C+O showed remarkable lethality of 50% and 60%, respectively. At 9 h, 2% O, 2% C+O, and 3% C+O remained at 10% lethality. In contrast, 3% O, 5% O, 7% O, and 5% C+O exhibited a lethality of 20%. The lethality for 7% C+O remained at 50% while it has increased for 10% O to 40% and for 10% C+O to 80%. By 12 h, lethality in oregano samples with concentrations below 7%, as well as 2% C+O and 3% C+O, remained unchanged. At this time, 7% O and 5% C+O demonstrated 30% lethality, while 10% O displayed 50% lethality. Lethality increased for 7% C+O to 60% and for 10% C+O to 90%. At 24 h, the lethality of 2% O and 2% C+O stayed at 10% and that of 3% O and 3% C+O stayed at 30%, while the lethality of 5% O increased to 30%. The 7% O, 10% O, and 5% C+O doses exhibited 50% lethality, 7% C+O remained at 60% lethality, while 10% C+O reached a maximum lethality of 100%. Finally, at 36 h, the lethality remained the same for all samples. In control (NA) and ethanol (ET) samples, all 10 weevils remained alive till the end of the experiment. This indicates that external factors and ethanol had no direct effect on the weevils’ viability.

The observed insecticidal activity may be linked to components like citronellol and citronellal, present in JCEO [46], as well as carvacrol, present in SOEO [47]. These compounds are characterized by a repellent effect against insects. The findings of this research align with those of other studies that affirm the toxicity of various EOs against weevils, including the EOs of thyme, anise, citronella, and rosemary [48]. Another study was conducted where *Citrus limonum* essential oil also had insecticidal activity against adult granary weevil and the efficacy of the EO depended on the duration of exposure [49]. Also, several species of *Cymbopogon*, including *C. winterianus,* were toxic to *Sitophilus oryzae* (rice weevil) [50], and OEO abundant in carvacrol was documented as a potential source of environmentally friendly insecticides to control insect pests [51].

## 4. Conclusions

This study’s findings effectively supported our objectives. A phytochemical screening revealed a significant presence of secondary metabolites, as well as elevated levels of total phenolics and flavonoids, alongside the antioxidant activity of Syrian oregano leaves. Our research elucidated the plant’s richness in antioxidants, which have the capacity to combat free radicals and mitigate disease risk. Furthermore, the incorporation of Syrian oregano, either alone or in combination with Java citronella, in powdered form into flatbread was well-received by consumers and demonstrated an extended shelf life compared to control flatbread by slowing down mold growth. The optimal concentration of 2% for the combined plant powders suggests a synergistic effect in their antioxidant preservative properties, surpassing the efficacy of oregano alone. Additionally, our findings indicate that Syrian oregano and Java citronella essential oils possess insecticidal potential, with 7% and 10% OEO + JCEO exhibiting the most effective treatments against *Sitophilus granaries* weevils. However, a lower concentration of 7% may be recommended due to the advantages of a reduced insecticide dose, alongside the time-consuming and costly nature of essential oil extraction. Our study aligns with observations that antioxidant-rich plants, such as oregano and orange, exhibit significant mold-inhibiting properties in bread. Previous studies also corroborate the insecticidal effects of essential oils from various medicinal plants, including thyme, anise, citronella, and rosemary. Based on these findings, further research is warranted to enhance the acceptability of flatbread supplemented with Syrian oregano and Java citronella, to investigate its antioxidant properties, and to optimize the final product for large-scale industrial applications, as well as to improve the output of these essential oils for insecticidal purposes.

## Figures and Tables

**Table 1:** Qualitative identification of primary and secondary metabolites in JCEO.

Metabolites	Extract	Reagent	Color	Reference
Reducing sugar	0.5 mL	1 mL water + 5–8 drops Fehling’s (A+B) + boiling (5 min)	Brick-red precipitate	[20]
Anthraquinones	1 mL	1 mL HCl (10%) + boiling (5 min)	Precipitate	[21]
Proteins and amino acids	1 mL	1 mL ninhydrin (0.25%) + boiling (5 min)	Blue color	[22]
Phlobatannins	1 mL	1 mL HCl (1%) + boiling (5 min) + cooling	Red precipitate	[23]
Tannins	1 mL	1 mL ferric chloride (FeCl_3_ 1%)	Blue color	[24]
Resins	1 mL	0.5 mL acetone + small amount of water + agitation	Turbidity	[25]
Terpenoids	1 mL	2 mL chloroform + 1 mL concentrated sulfuric acid	Reddish brown color on the surface	[20]
Flavonoids	1 mL	5 mL potassium hydroxide (KOH 50%)	Yellow color	[26]
Quinones	1 mL	1 mL HCl concentrated	Precipitate or yellow color	[21]
Sterols and steroids	1 mL	2 mL chloroform + 1 mL concentrated sulfuric acid	Red color of the upper layer + greenish yellow fluorescence in the acid layer	[22,27]
Diterpenes	1 mL	Few drops of copper acetate	Green color	[24]
Anthocyanins	1 mL	1 mL NaOH (10%)	Blue color	[28]
Flavanones	1 mL	${1mLH}_{2}{SO}_{4}$ concentrated	Purple red color	[28]
Cardiac glycosides	2 mL	$1\;\mathrm{mL}\;\mathrm{acetic}\;\mathrm{acid}\;\mathrm{glacial}+1\;\mathrm{drop}\;\mathrm{ferric}\;\mathrm{chloride}\;({FeCl}_{3}$ 5%) + 1 mL concentrated sulfuric acid	Purple ring + Brown ring + Green ring	[22,28]
Saponins	2 mL	Vigorous shaking (5 min on vortex)	Layer of foam	[20]
Phenols	5 mL	$1\;\mathrm{mL}\;{FeCl}_{3}$ (1%) + ${1mLK}_{3}{\left(Fe(CN\right)}_{6}$) (1%)	Greenish blue color	[24]
Fixed oils and fatty acids	Small amount of extract	On filter paper	Oil spot	[20]

**Table 2:** Flatbread sample codes and composition.

Sample Code	Syrian Oregano Powder (%)	Java Citronella Powder (%)
C	-	-
0.5O	0.5	-
1O	1	-
1.5O	1.5	-
2O	2	-
2.5O	2.5	-
3O	3	-
0.5C+O	0.25	0.25
1C+O	0.5	0.5
1.5C+O	0.75	0.75
2C+O	1	1
2.5C+O	1.25	1.25
3C+O	1.5	1.5

-: Absence.

**Table 3:** Petri dish sample codes.

Sample Code	SOEO (µL)	JCEO (µL)	Ethanol (µL)
NA	-	-	-
ET	-	-	1000
2% O	20	-	980
3% O	30	-	970
5% O	50	-	950
7% O	70	-	930
10% O	100	-	900
2% C+O	10	10	980
3% C+O	15	15	970
5% C+O	25	25	950
7% C+O	35	35	930
10% C+O	50	50	900

-: Absence.

**Table 4:** Chemical composition of the essential oil of *Origanum syriacum* leaves.

Peak	Compound Name	Retention Time (min)	Area Percentage (%)
1	α-thujene	4.73	1.65
2	4-carene	5.37	2.49
3	p-cymene	5.64	5.31
4	Cyclohexene	5.69	0.56
5	γ-Terpinene	6.69	9.77
6	cis-β-terpineol	6.82	0.46
7	Thymol	15.47	0.47
8	Carvacrol	17.75	79.30

**Table 5:** Phytochemicals of oregano extracts. (+: low presence; ++: moderate presence; +++: high presence; -: absent).

Metabolites	Aqueous Extract	Ethanolic Extract
Reducing sugars	++	-
Anthraquinones	+	+
Proteins and amino acids	-	++
Phlobatannins	+	+
Tannins	++	++
Resins	++	++
Terpenoids	++	++
Flavonoids	+++	+++
Quinones	++	++
Sterols and steroids	-	++
Diterpenes	-	++
Anthocyanins	-	-
Flavanones	++	++
Cardiac glycosides	++	++
Saponins	++	++
Phenols	++	++
Fixed oils and fatty acids	++	++

**Table 6:** LT_50_ of SOEO and SOEO+JCEO at different percentages.

Essential Oil Concentration	LT_50_
2% O	NA
3% O	NA
5% O	NA
7% O	24 h
10% O	12 h
2% C+O	NA
3% C+O	NA
5% C+O	24 h
7% C+O	6 h
10% C+O	Between 30 min and 6 h

NA: not applicable—LT_50_ was not attained.

## Data Availability

The original contributions presented in this study are included in the article. Further inquiries can be directed to the corresponding authors.
